# Framework for Structural Health Monitoring of Steel Bridges by Computer Vision

**DOI:** 10.3390/s20030700

**Published:** 2020-01-27

**Authors:** Adam Marchewka, Patryk Ziółkowski, Victor Aguilar-Vidal

**Affiliations:** 1 Computer Science and Electrical Engineering, Faculty of Telecommunications, University of Science and Technology in Bydgoszcz, Al. prof. S. Kaliskiego 7, 85-796 Bydgoszcz, Poland; adimar@utp.edu.pl; 2 Faculty of Civil and Environmental Engineering, Gdansk University of Technology, Gabriela Narutowicza 11/12, 80-233 Gdansk, Poland; 3 Department of Civil Engineering, Auburn University, 261 W Magnolia Ave, Auburn, AL 36849, USA; vha0001@auburn.edu; 4 Facultad de Ingeniería y Tecnología, Universidad San Sebastián, Lientur 1457, Concepción 4080871, Chile

**Keywords:** computer vision, drones, image processing, steel structures, structural health monitoring

## Abstract

The monitoring of a structural condition of steel bridges is an important issue. Good condition of infrastructure facilities ensures the safety and economic well-being of society. At the same time, due to the continuous development, rising wealth of the society and socio-economic integration of countries, the number of infrastructural objects is growing. Therefore, there is a need to introduce an easy-to-use and relatively low-cost method of bridge diagnostics. We can achieve these benefits by the use of Unmanned Aerial Vehicle-Based Remote Sensing and Digital Image Processing. In our study, we present a state-of-the-art framework for Structural Health Monitoring of steel bridges that involves literature review on steel bridges health monitoring, drone route planning, image acquisition, identification of visual markers that may indicate a poor condition of the structure and determining the scope of applicability. The presented framework of image processing procedure is suitable for diagnostics of steel truss riveted bridges. In our considerations, we used photographic documentation of the Fitzpatrick Bridge located in Tallassee, Alabama, USA.

## 1. Introduction

Structural safety is one of the most critical issues during the life cycle of constructions, especially in bridges [1,2]. Bridges carry traffic loads that are steadily increasing in volume and weight. They are subjected to deterioration due to ageing, as well as corrosion, fatigue of materials, extreme environmental loads, and unexpected impacts. As a result, damage to the bridge superstructure or substructure may occur, such as cracking, delamination, loss of cross section, foundation settlement and others [3,4]. Those adversely affect a structural performance, safety, reliability and they are potentially harmful. The damage of a bridge can have significant economic impacts and quickly can become a disaster. Although the importance of bridges is not under discussion, multiple complications make the inspection and monitoring of bridges a time-consuming and expensive task [5,6]. Moreover, there is often a lack of funds for inspections, maintenance, and repairing work. Therefore, there is a need for cost-effective solutions for inspection that allow for rational prioritization of maintenance and repairs, so resources are efficiently assigned. 

Structural health monitoring could safeguard the safety and reliability of our infrastructure while reducing maintenance costs, making inspection faster and more cost-effective [7,8]. The primary purpose of the structural health monitoring system is to keep and improve its safety. The assessment of the structural condition of bridges and other related infrastructure utilising noninvasive monitoring techniques is an excellent promoter of emerging new technologies, and the field gained significantly by recent developments. For example, currently, there is a wide variety of affordable, robust sensors for harsh environments, highly reliable wireless networks, the possibility of store data in the cloud, high-speed connection that allows real-time monitoring and alert systems and the availability of previously unseen high-resolution cameras, are some of the latest technological improvements that are rapidly changing the structural health monitoring field [9]. 

**Figure 1:**
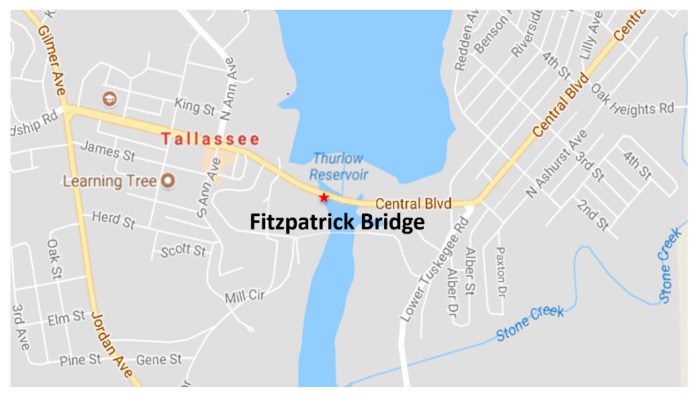
Location of Fitzpatrick Bridge.

**Figure 2:**
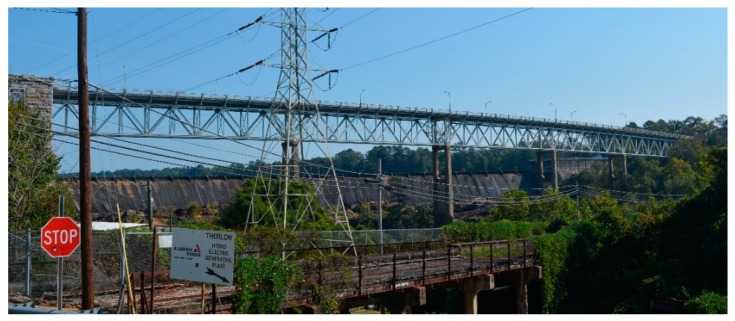
Fitzpatrick Bridge side view.

A well-designed structural health monitoring system should provide information about the condition of the structure and alert the owner of this construction about problems or poor technical conditions, which may lead to failure and, as a consequence, to a potential disaster [10]. We believe that the decision-maker who uses the system should receive information about the behaviour of structures and situations that require intervention or involve the risk of exceeding the load-carrying capacity considered as safe. In this paper, we propose the framework for construction monitoring system based on autonomous measurements performed using the Unmanned Aerial Vehicle (UAV) and a Digital Image Processing (DIP). In our analysis, we used the photographic documentation of the Fitzpatrick Bridge located in the city of Tallassee, Alabama, in the United States. The considered case of study is a riveted steel truss bridge supported on concrete pylons. Figure 1 presents the location of the Fitzpatrick Bridge, and Figure 2 shows the side view of the span.

## 2. Steel Bridge Structural Health Monitoring

### 2.1. The Issues of Monitoring Steel Bridges

Currently used structural health monitoring systems can be defined as a set of interconnected devices and dedicated software that continuously perform measurements of structure behaviour, archive and analyse the results of these measurements. Therefore, a typical system consists of a measuring instrument installed on the structure and computing module, generally computer with dedicated software, responsible for the archiving and results analysis, which we can install outside of the structure. The structural health monitoring system, depending on the needs, size and type of the structure, should perform measurements with the frequency from fractions of seconds to several hours or days. The system also should be efficient and durable. The importance of structural health monitoring issues is emphasized by the introducing, in 2009, of a European legal act “Regulation of the Minister for Infrastructure—12 March 2009, amending the regulation on technical conditions that should be met by buildings and their location” (Journal of Laws from 2009 No. 56, item 461, §204, paragraph 7) requirements for constant monitoring of essential parameters for the safety of structures, such as displacements, deformations and stresses, in public utility structures, including public entertainment arenas, sports arenas, public transportation stations, exhibition halls and commercial buildings. This regulation does not apply to the most ordinary buildings with steel structures, such as logistics centres, warehouses and industrial halls. In our method, we will not make exact measurements of displacements, stresses and strains, but instead, we would like to use computer vision to analyse the image and capture visual markers that indicate a poor structural condition or pre-failure state of the structure.

### 2.2. Monitoring of Steel Bridge Constructions Using Computer Vision

The subject of structural health monitoring by computer vision is widely commented on in the current scientific discourse. Feng et al. [11] used sub-pixel template matching as a tool for vision-based displacement measurement method. The method involves the use of Fourier transform for upsampled cross-correlation. Their field test consists of shaking table and bridge field tests. They achieved ±1 mm accuracy in measuring structural vibrations. Ye et al. [12] analysed vision-based structural displacement measurement method and evaluated its performance. They integrated the DIP with a vision-based structural displacement measurement system. Their study revealed that illumination and vapour could significantly affect the test results. A fascinating application of vision-based structural health monitoring application is one presented by Vicente et al. [13] with the use of laser beam, LED light source and video camera to measure static vertical deflections of the bridge. Schumacher and Shariati [14] proposed a method that uses video-based tracking of designed sensors placed on the deck. A different look at bridge structural health monitoring presents Zhao et al. [15], where they used Laser Projection-Sensing Technology. Abdel-Qader et al. [16] use a region growing-based approach to segment thermal images for noninvasive evaluation of bridge decks. The segmentation algorithm begins with the one pixel in the image as seed points, which represents the hottest area and then regions are grown around them based on a neighbourhood selection criterion. Sharifzadeh M. et al. [17] developed a method for detection and classification of steel surface defects. Feng and Feng [18] confirm that diagnostic systems based on digital image analysis are cost-effective and present their findings on the example of Manhattan Bridge. In their considerations, they analysed displacements based on the bridge image for various points in time. Lee et al. [19] presented an adaptive Region of Interest (ROI) process for target marker detection. They validated it by a laboratory, with artificially generated light and field tests during sunlight. They also claim that their approach is resilient towards changing light conditions. 

### 2.3. Bridge Inspection Standards and Visual Markers Indicating Deterioration of Structural Elements

#### 2.3.1. Markers of Structural Deterioration Feasible for Visual Inspections

**Figure 3:**
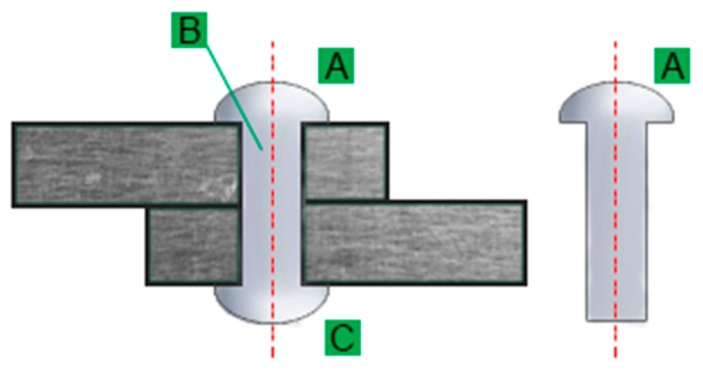
A rivet cross section: (A) head, (B) shank and (C) tail.

To identify damages and pre-emergency conditions, we must define which visual markers will indicate a poor condition of the structure. For this purpose, we analysed modes of failures in cross-braced steel bridges. In the considered case, the rivets connect diagonal sheaves. Before being incorporated into the structure, the rivet consists of a head and a shank, as in Figure 3. After inserting the elements to be joined in the hole, it is coiled in the swelling process of the spindle, and a strike closes it. The swelling of the spindle is usually performed by hammer or a riveting tool. The length of the mandrel must include at least the length of the joined elements with a specific allowance necessary to form the ferrule.

**Figure 4:**
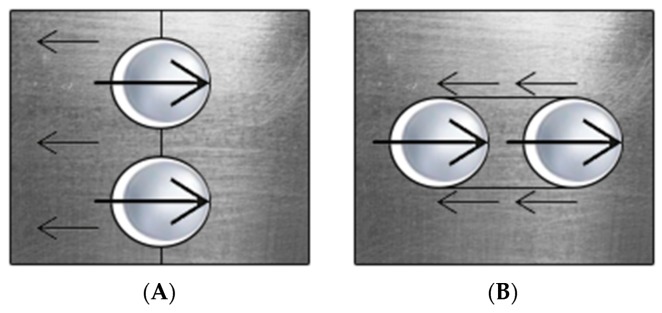

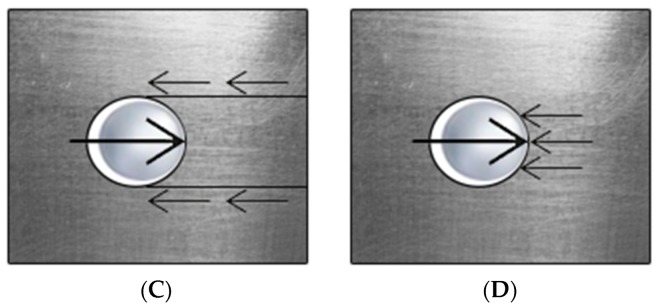
Modes of failure associated with pressure of the rivet on metal sheet: (**A**) cutting metal sheets between holes in the direction of load, (**B**) cutting metal sheets between the hole and the edge of the metal sheet in the load direction, (**C**) tearing the metal sheets in a direction perpendicular to the effort axis, and (**D**) reaching bearing capacity due to the pressure of the rivet mandrel to the hole.

The riveting process can be carried out both hot and cold, where bridge rivets are hot-rolled. Steel girders, which are joined by rivets, are usually drilled to form holes. Depending on the girders’ thickness, pneumatic plungers are also used to puncture the holes. It is essential to know that even though the described connection is a partially friction one, in the calculations, it is assumed that the rivets transfer the entire load. The rivets transfer tension and shear forces. In the considered bridge trusses, we can distinguish several modes of failure that can determine how we will analyse the image. Steel girders can reach its bearing capacity due to various modes, such as rapture of joined sheets or overlays, tearing out rivets from a metal sheet by shearing of metal sheet or rivets, detachment of rivet head, buckling of compressed rods and deformation of rivet holes or rivets [20,21,22]. There are also other critical modes, associated with the pressure of the rivet on metal sheet [21], such as cutting metal sheets between holes in the direction of load, cutting metal sheets between the hole and the edge of the metal sheet in the load direction, tearing the metal sheets in a direction perpendicular to the effort axis, reaching bearing capacity due to the pressure of the rivet mandrel to the hole. Figure 4 presents the modes of failure mentioned above.

**Figure 5:**
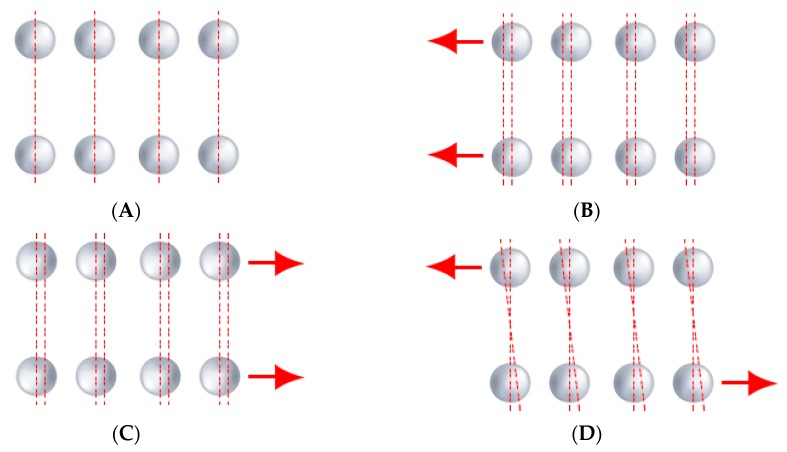
Local displacement of rivets: (**A**) original image of riveted steel girder, (**B**) displacement of rivets towards the node, (**C**) displacement of rivets away from the node, and (**D**) displacement of rivets in opposite directions.

Small displacements will proceed discussed modes of failures so that we can identify and track them. In our opinion, we can achieve that by tracking the displacement of extracted rivet pairs in separate bridge brace. A significant change in the position of the rivets pairs to each other over time may indicate a pre-emergency condition. Figure 5 presents observable changes in the position of the rivets for the separated cross-brace of the steel bridge.

**Figure 6:**
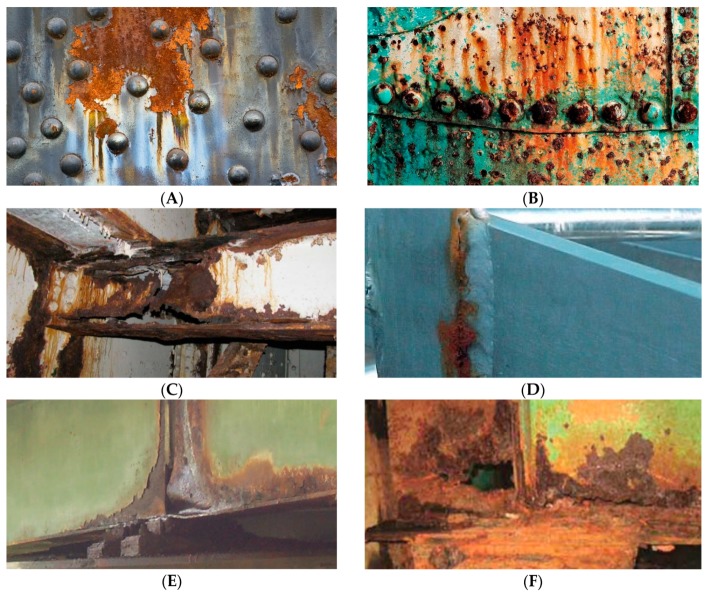
Visual markers indicate the poor condition of the structure: (**A**) visible corrosion of steel around the rivets, (**B**) visible corrosion of rivets, (**C**) visible defects in the material due to corrosion in joint, (**D**) visible corrosion of the weld and (**E**,**F**) visible defects at the supports due to corrosion [24,25].

Another feature that may indicate a poor condition of the structure is the extensive corrosion of both the plate girders and the rivets. Excessive corrosion can lead to a reduction in the effective cross-section and, consequently, to failure [20,23]. Areas at particular risk are in stress concentration zones [23], such as joints and supports [24,25]. In Figure 6, we illustrated visual corrosion-related markers indicating a poor condition of the structure.

## 3. Materials and Methods

### 3.1. Essentials

**Figure 7:**
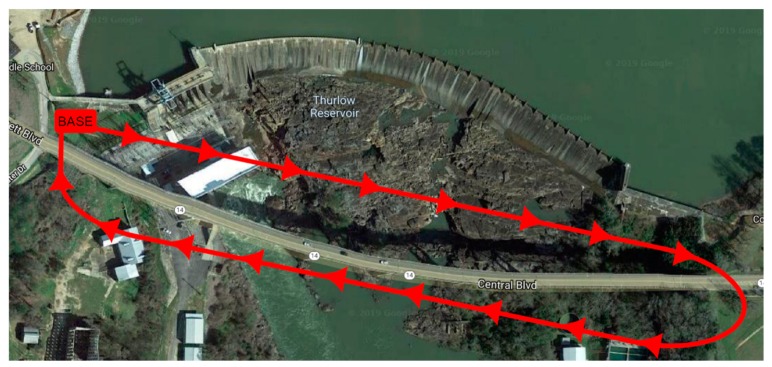
Fitzpatrick bridge in top view, in a straight-line bridge, is 492 m (1614.20 ft.) long.

Recently, in search of financial savings as well as human resources, local Departments of Transportation in the United States began to consider using drones to perform bridge inspections [26]. The American Association of State Highway and Transportation Officials March 2016 survey found that 17 state Departments of Transportation had studied or used drones, whereas 16 states were either exploring UAV usage, assisting in the development of drone policies, or supporting drone research [27,28,29]. UAVs themselves cannot perform inspections independently, but can be used as a tool for bridge inspectors to view and assess bridge element conditions following the National Bridge Inspection Standard [30,31,32]. We believe that to achieve profits resulting from the automation of the image analysis process, it is also essential to automate the process of data acquisition, which is the drone flight route. We recommend programming the drone to perform a predetermined path around the bridge, which, in the case of a large number of bridge objects, will allow for significant automation of work and potentially can bring considerable savings [33]. Figure 7 presents an example drone flight route for the case of the bridge we are considering in this study.

**Figure 8:**
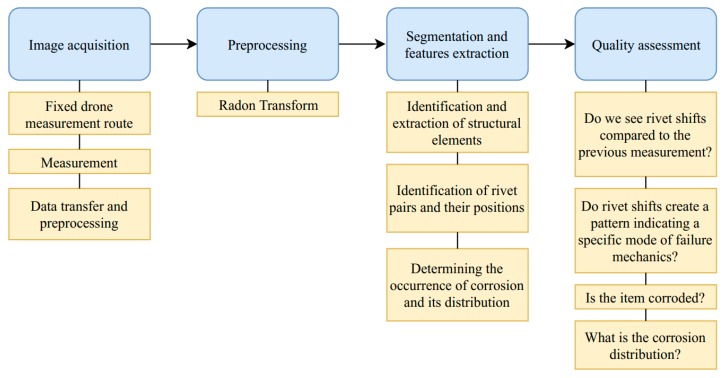
Outline of the monitoring process.

The UAV flight route should not run above the road, so that in the event of a breakdown, it does not fall onto the road surface and cause an accident. The drone route should also be straight that, on the one hand, it will reduce the energy consumption of the equipment and, to some extent, prevent the device from rocking, which would negatively affect the collected material [34,35,36]. A unique way to plan a drone flight route was presented by Sunghun Jung [37], in which he showed an algorithm that provides a flight path with low electricity consumption. Ultimately, to limit any human interference, we recommend installing a permanent UAV nest near the bridge, from which the drone would take off and in which it could recharge a battery. We believe that such an installation for constant monitoring could be an element determining the reliability of the structure. Correct data acquisition is a crucial aspect of the entire monitoring process [38]. The requirements that are set during image acquisition are closely related to the needs that define the accuracy of the measured changes. This means that during image acquisition, there are several important issues, such as appropriate selection of the camera focal length-blurred image makes it difficult to perform correct estimates by the proposed method [39], appropriate close-ups of details the accuracy of measurements are directly proportional to the number of image points (pixels) representing one millimetre of the real object (accuracy of 1 mm/number of pixels) [40], orthogonality of the optical axis to the tested object and elimination of external factors such as atmospheric conditions, snow, rain, or sun that cause glare or shadow. The study on visual markers and modes of failure prompted us to conclude possible solutions, which allow as identifying the deteriorating condition of the structure efficiently. We decided to focus on detecting rivets and their position, as well as determining the level of corrosion and its distribution. The bridge we analyse is a steel truss bridge. The first step our algorithm will perform is identifying and separating steel beams. Next, we will classify and extract the position of the rivets. In our view, by comparing the images in time, we will be able to determine if the position of the rivets has shifted relative to the previous measurement, and also what is the nature of this shift. Next, we will identify corroded places and determine their size. In Figure 8, we present a proposed multidetector system for monitoring steel-braced bridges. The entire monitoring process consists of four main stages, namely image acquisition, preprocessing, segmentation, features extraction, and quality assessment. Our unique contribution lies in merging computer science with structural engineering knowledge, to create a solution tailored for needs of structural health monitoring of steel bridges.

### 3.2. Method Application

The first stage is image acquisition of the monitored object, which should be performed by UAV, with favourable weather conditions, following the previously determined track. We described track selection and appropriate weather conditions in the previous chapter. As we mentioned before, it is essential to obtain good image quality. Corrupted visual material, for example, noisy or blurred images, will not be suitable for processing in the next stage. The image obtained during the acquisition process must first be appropriately processed. This process is called preprocessing, and it is designed to eliminate unwanted phenomena that may occur during the image acquisition. It is vital to remove these anomalies in the initial phase due to error propagation in further stages. The most common problems are electronic noise or geometric deformations resulting from the use of a particular type of lens. The use of appropriate filters allows reduction of the occurring noise, and the calibration process has a positive effect on improving the geometry of the image. However, great caution should be exercised, because each of the above techniques can negatively affect the results of the proposed method. In further image analysis, we will often refer to the edge image. Such an image is obtained by using an appropriate edge detector algorithm, such as Prewitt, Sobel or Canny Edge Detector. The third stage consists of extracting the elements of particular interest and determining their parameters. This approach can be described as a general-to-specific order. Therefore, first, we extract bridge structural elements, in our case steel beams, and then within the extracted image, we look for rivet heads. We will use the extracted rivet positions to determine their centres of gravity and, subsequently, estimate their exact location. Thanks to this, we will be able to decide whether their position has changed and its character. Typically, these operations are performed manually by the system operator, which is tedious and lengthy.

For purposes of segmentation, utilizing given characteristics of the tested object, as for us longitudinal steel beam, we decided to use the Radon Transform [41]. Equation (1) defines the Radon transform.

(1) $$R_{\theta }\left(\rho \right)=\overset{\infty }{\underset{-\infty }{\int }}f\left(x^{\prime }cos\theta -y^{\prime }sin\theta ,x^{\prime }sin\theta +y^{\prime }cos\theta \right)dy^{\prime }$$

where ρ is a distance from the centre of the line, θ is an angle of the normal vector and x and y are coordinates of the image point. A straight-line at an angle θ−90° and distant from the centre of the image by a distance ρ in Radon space is marked as the local minimum. Over the years, it has been proven that this mathematical apparatus works excellently at detecting straight lines in the image. It is the parallel straight lines that can define beams in the image. To eliminate incorrect indications and to ensure adequate accuracy of classification, we assumed that a beam meeting the criteria in the Radon space, as defined in Equation (2).

(2) $$\left\{R_{\theta }\left(\rho_{k}\right),\;R_{\theta }\left(\rho_{l}\right)|\left|\rho_{k}-\rho_{l}\right|\geq d\right\}\;$$

where $R_{\theta }\left(\rho_{k}\right),\;R_{\theta }\left(\rho_{l}\right)$ are the local minimum and d is the minimal beam thickness. The d value is directly related to the accuracy of the measurements. This correlation can be defined as in Equation (3).

(3) d=dzq 

where $d_{z}$ is the actual width of the beam and q is a maximum absolute error. A condition contained in Equation (2) guarantees that the lines defining the beam are parallel to each other and thus perpendicular to the observer; consequently eliminating the influence of geometric distortions on the further image analysis process. 

Next, we perform a feature extraction, which we use for rivet detection. In such a limited search area, we want to determine the position of the individual centres of gravity of the rivet heads. To serve this purpose, we subject the edge image to morphological operations such as Erosion and Dilation [42,43]. The use of these two filters helps us to eliminate unnecessary elements from the edge image. Then, we can detect individual objects separated from each other and determine their geometrical features by introducing restrictions such as in Equation (4).

(4) $$S=\;\left\{s\left(w,h\right)|w\geq d_{1,}w\leq d_{2,}h\geq d_{1,}h\leq d_{2,}\right\}$$

where s(w,h) is a sought object, w is a width of the object, h is the object height, and $d_{1,}d_{2,}$ are threshold values for the rivet size and are directly related to the beam thickness d. Based on the change in the centre of gravity of the elements in the set S, we can estimate the nature of the change in the structural element. 

Afterwards, we estimate areas of the monitored object affected by visible corrosion. The criterion is a colour represented in YCbCr space [44,45]. Simple colour thresholding may prove to be ineffective. Therefore, we propose to adapt the model usually used for skin detection [46]. To carry out this, we built a rust base, a histogram of information about the colour in the form of two chrominances $Y_{r},\;Y_{b}$. Then, we approximate n histogram maxima by the two-dimensional Gauss function, as in Equation (5).

(5) $$G(Y_{r},\;Y_{b})=\;\frac{1}{2\pi \alpha^{2}}e^{\frac{Y_{r}^{2}+Y_{b}^{2}}{2\alpha^{2}}}$$

where α is a smoothing parameter of the function; this method allows us to identify regions potentially attacked by rust. 

So far, we focused on processing and extracting relevant data from images. The last part of our consideration is building proper quality assessment classifiers. The first criterions concern the behaviour of steel structural elements, which we assume that can be observed by tracking the position changes. Observation can be divided into two categories: macro-observation and local-observation. Macro-observation concerns the investigation of the position of structural elements in the scale of the entire object, which, in our opinion, will be more accurate because we can track emerging trends and even if the position of some elements is read with an error, their large number should not disturb results. To observe an object on a macro-scale, we should observe changes in the position of the structural elements, relative to the overall deflection line of the static system, which allow us to identify areas that can be subjected to the largest displacements. For example, when considering a simply-supported beam loaded with an evenly distributed load, we will be interested in the displacement of the span area in relation to the supports.

On the other hand, local-observation gives us a clue to what happens to the structural element concerning its immediate surroundings, which will allow us to identify progressive local distortions, such as lateral-torsional buckling. Detection of rust spots and its distribution might be a puzzling issue, as not every discolouration indicates rust and not every rust area implies danger to the construction. To tackle discolouration we built a unique colour model of rust that is discussed in details in Section 4. Rust will be the most dangerous in areas of highest stress concentration, such as supports, we have discussed more visual markers in Section 2.3.1. We have shown illustrations of corroded areas in Figure 6.

### 3.3. Proposed Methodology

**Figure 9:**
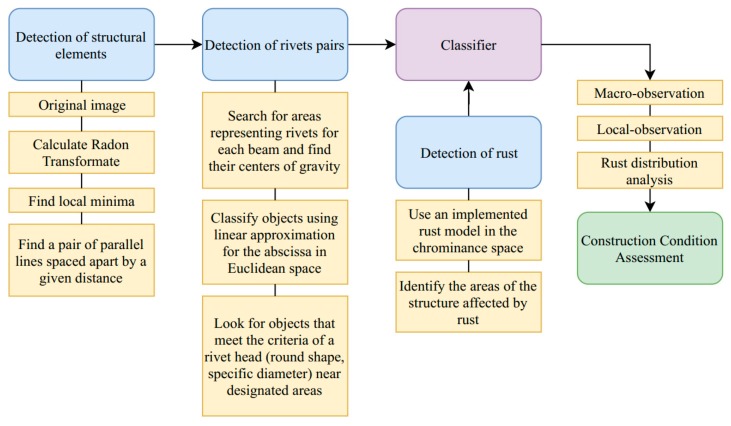
Multi-detector system in details.

To sum up our consideration on the multi-detector system we presented a detailed solution in Figure 9.

## 4. Results and Discussion

In this chapter, we present the results of implementing the method we developed on the example of considered case of study, the Fitzpatrick Bridge.

### 4.1. Image Acquisition

As we emphasised, the image acquisition procedure is one of the most significant ones in the entire monitoring process, and it is directly connected with our beam detector. Therefore, we believe that the operator should be notified in real-time about the initial quality verification of the images of the observed object. In our case, the maximum measurement error is the minimum value of the tested distortions and is equal to 1 mm. If the algorithm measures distortion with an accuracy of one image point, then for a beam whose actual width is $d_{z}$ = 220 mm. Therefore, its width in the image must be represented by 220 or more image points. Compliance with this condition allows further analysis of the image. We used a camera with the following features; aperture unit: 5.6; focal length: 300 mm. Besides, the image had the following parameters: vertical and horizontal resolution: 300 dpi; depth in bits: 24; colour reproduction: sRGB. Images were saved in JPG format with compressed bits per pixel equal 4. The choice of compression level was conscious. It is known that during image compression, we irrevocably lose some of the information contained in it, and this can negatively affect the classification process. However, we obtained satisfactory results so we did not investigate it further. We must also point out that the images were taken during a sunny day. Restricting access to light or taking photos at night would certainly not allow you to obtain the material needed for research. However, to solve this problem, we could use a flash during acquisition, as is done during pavement distortion measurements [1]. The use of the flash would eliminate the shadow effect (which by the way our algorithm deals with satisfactory), and would achieve a significant improvement, especially in the rust segmentation.

### 4.2. Beam Detection

**Figure 10:**
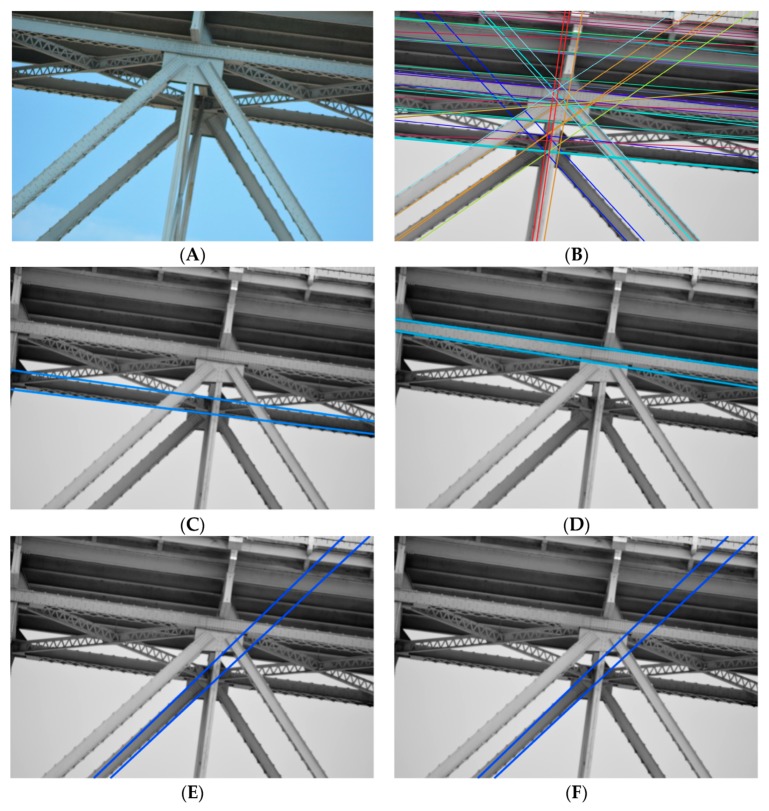
(**A**) Original image. (**B**) Preliminary beam identification. (**C**–**E**) Correct beam detection. (**F**) Incorrect beam detection.

The steel beam segmentation technique is based on the use of Radon transform properties. We adopted several assumptions that must be met during image acquisition to achieve proper results during the implementation of the method. We made a series of tests on the sample images. We checked whether the image elements meet the criterion (2). In Figure 10, we presented both original and an image with preliminary beam identification, as well as an illustration of how the algorithm works for particular beams. We obtained quite good results for beam segmentation. However, some beams were classified incorrectly, as shown in Figure 10D.

### 4.3. Rivet Detection and Displacement Tracking

**Figure 11:**
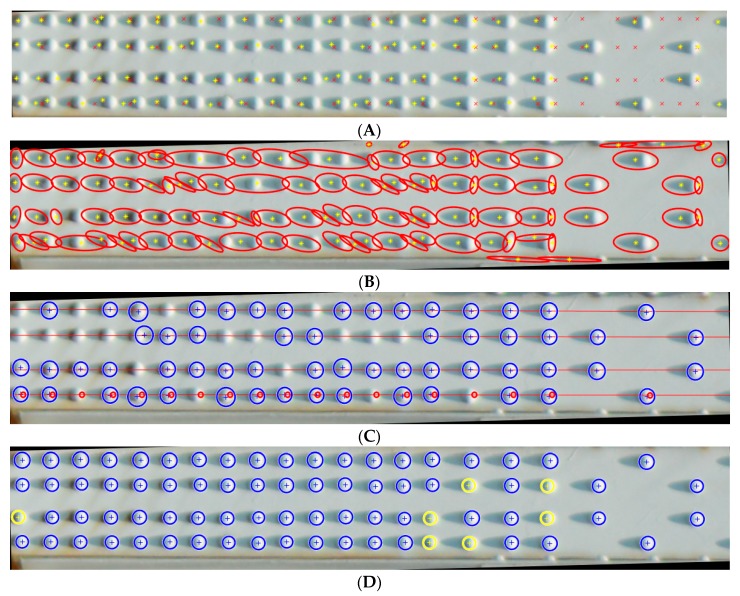
Rivet detection: (**A**,**B**) Search for areas representing rivets for each beam and find their centres of gravity. (**C**) Classify objects using linear approximation for the abscissa in Euclidean space. (**D**) Look for objects that meet the criteria of a rivet head (round shape, specific diameter) near the designated straight lines.

We detected rivet heads with the determination of their geometrical position relative to each other for each extracted beam. We determined the criterion for segmentation of rivet heads based on the assumptions, as follows, each head of the rivet has a round shape, the rivets are arranged systematically, and the distance between the rivets must allow the identification of the circular shape of the rivet. Our algorithm consists of several parts. Figure 11 presents the execution of the algorithm. Based on the edge image properties, we perform objects segmentation (yellow points: Figure 11A) and estimate the rivet pitch (red points: Figure 11A). We search for areas representing rivets for each beam and find a circular shape and their centres of gravity in the designated areas [47,48], as in Figure 11B. Next, we classify objects using a linear approximation for the abscissa in Euclidean space, as in Figure 11C. Eventually, we look for objects that meet the criteria of a rivet head (round shape, specific diameter) near the designated straight lines, as in Figure 11D. Blue circles are detected rivets, whereas yellow circles indicate the probable occurrence of the rivet head. Yellow circles are probable locations: due to excessive blurriness of the image, it was not possible to extract the edge of the rivet head. Our algorithm in the analysed image (Figure 11) of the separated steel cross-braced beam of the bridge correctly detected 71 rivets (92%) and correctly determined the probable position of the remaining 7 (8%). We analysed 12 images of the segmented steel cross-braced beam, achieving on average 97.2% correctly detected rivets, 1.3% incorrectly detected rivets and 0.9% undetected rivets.

**Figure 12:**
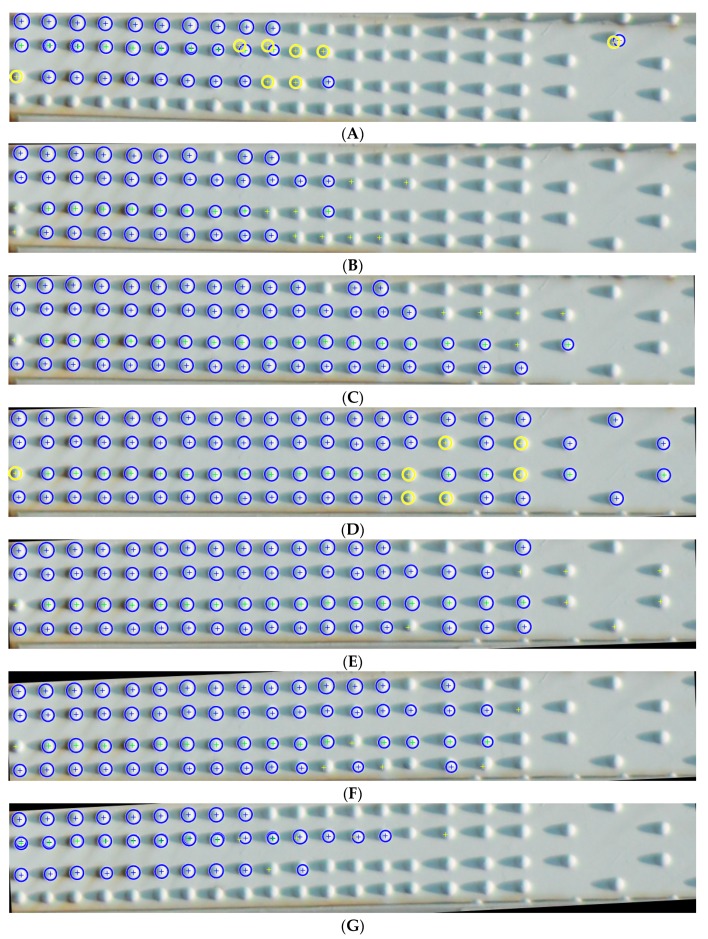
Rivet detection in relation to rotation angle of the image: (**A**) θ = −1.5°, (**B**) θ = −1.0°, (**C**) θ = −0.5°, (**D**) θ = 0.0°, (**E**) θ = 0.5°; (**F**) θ = 1.0°; (**G**) θ = 1.5°.

In the next analysis, we examine the robustness of the developed method for an un-segmented or poorly segmented image. For this purpose, we performed an analysis in which we rotate the image of the beam by a given angle and detect rivets, as presented in Figure 12.

We also check the deviation of the Euclidean distance of the rivet heads from the reference point depending on the rotation of the image, as shown in Table 1.

Based on our analyses, we concluded that the measurement error is within one image point. In our case, one image point corresponds to one millimetre. Despite the image rotation, the rivet head detection itself falls within the error tolerance. Our robustness test confirmed that the rotation of the image directly affects the number of rivet heads found. It means that the proposed method is susceptible to poor beam detection, where even a 0.5 degree rotation can reduce the efficiency of the method by 20% for a single beam. Based on the robustness test of our solution, we can assume that it is necessary to use the beam segmentation algorithm that aligns the beam image despite the variable position resulting from the arrangement in the structure as well as disturbances in image acquisition. We also conclude that the image acquisition should be carried out at a frequency of 25 frames per second, which allows up to 25 measurement samples per second in changing conditions during drone flight. In further research, we plan to carry out a series of drone flights for different objects and at different time intervals, which we were not able to perform within this study.

**Figure 13:**
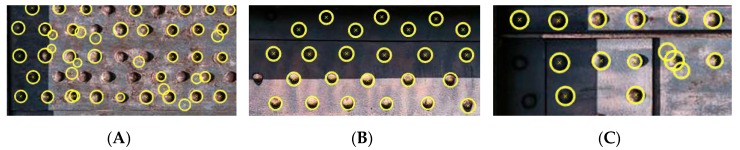
Clippings from sample results of the additional analysis: (**A**) The most unfavourable test result—size 295 × 158, correctly detected rivets 80%, incorrectly detected rivets 20% and undetected rivets 37%; (**B**) Size 320 × 135, correctly detected rivets 96.6%, incorrectly detected rivets 3.3% and undetected rivets 0%; (**C**) Size 395 × 95, correctly detected rivets 88.5%,, incorrectly detected rivets 11.5% and undetected rivets 26.9%.

However, to check the operation of the algorithm for objects other than considered bridge, we obtained a number of images from publicly available sources and perform additional tests. To make the task more difficult, we picked up images with a high compression ratio and very corroded surfaces. For 20 analysed images, we achieved on average 79.5% correctly detected rivets, 30.6% incorrectly detected rivets and 21.1% undetected rivets. We presented clippings from sample results of the additional analysis in Figure 13.

### 4.4. Rust Detection

**Figure 14:**
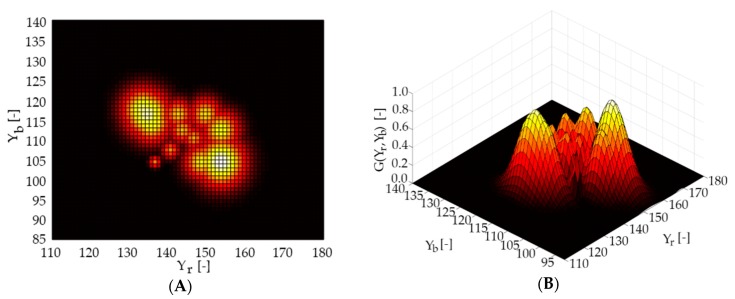
Distribution of rust colour pixels in YCbCr space after multiple Gaussian approximations. (**A**) Yb-Yr, two-dimensional view. (**B**) Yb-Yr- G(Yr,Yb), three-dimensional view. Yb and Yr is the blue element and red element related to the chroma element, which means that Yb is the blue element relative to the green element. Yr is the red element corresponding to the green element. G(Yr,Yb) is the luma component of the colour, which its brightness.

**Figure 15:**
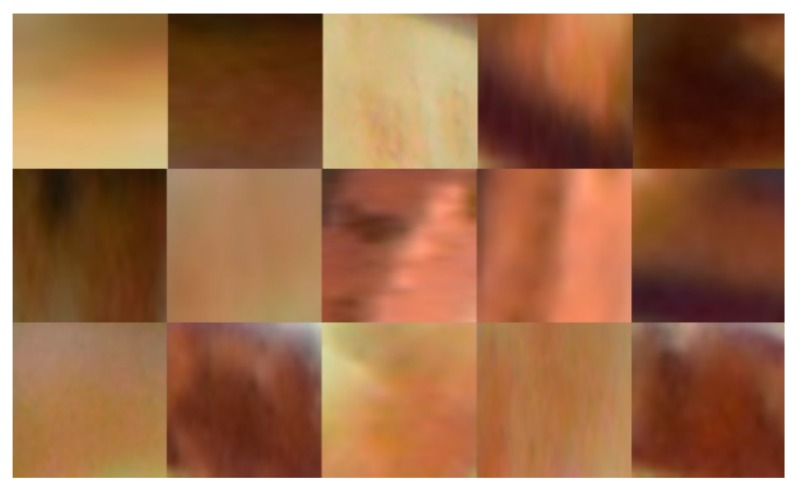
A database of rust colour pixels.

**Figure 16:**
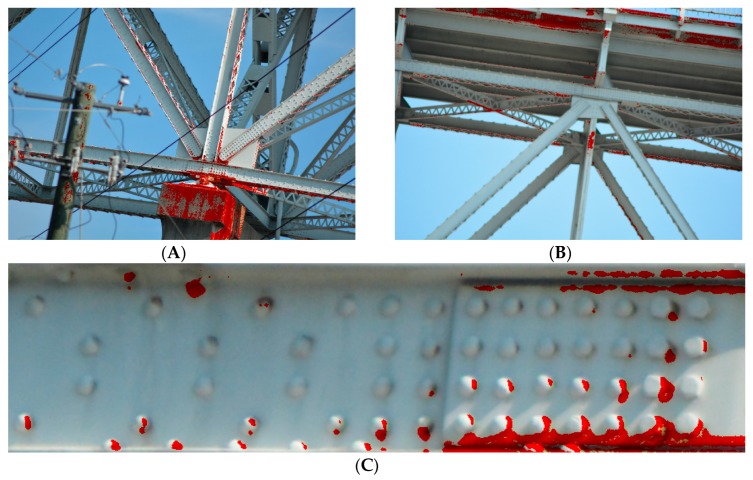
Rust detection: (**A**,**B**) close-up to nodes and (**C**) close-up to rivets.

We built a rust colour model, as shown in Figure 14, to detect areas covered by visible signs of rust. The model we made is in the chrominance space based on 15 random samples representing the rusted area, as in Figure 15. The use of the mentioned model allowed us to correctly classify 96% of rust areas. Figure 16 shows the results of the rust detection and segmentation algorithm. The algorithm also mistakenly indicated some places where there was no rust. Note that the bridge support made of concrete gives false indications, as in Figure 16A. Therefore, we recommend performing segmentation before rust detection. As we presented, the rust classifier is based on the chrominance distribution of the image. Any change in lighting has a significant impact on this distribution. A compromise that gives satisfactory results is to build a distribution based on any test images using the Gauss function. The results can be also improved by using the flash and generating a dedicated rust chrominance distribution for a given object.

### 4.5. Discussion

We described the results from structural components segmentation, rivet detection and visual rust assessment for the considered case. In this chapter, we will analyse the presented framework, interpret the obtained results and consider the solution in compare to existing methods described in the literature. The framework of the solution that we proposed to perform UAV measurement flight route maintains safety standards and ensures an optimal level of energy consumption, but the accuracy of the data collected is very susceptible to many external factors. In terms of accuracy, continuous measurements will undoubtedly have the upper hand. As we mentioned in Section 2.2, Schumacher and Shariati [14] proposed a method that uses video-based tracking of designed sensors placed on the deck. Their feasibility study revealed that their approach could be accurately computed. However, the sensor that they used seems to be very fragile and susceptible to the adverse effects of weather conditions. There was no information about in-field tests. On the other hand, there is the method also mentioned in Section 2.2 by Zhao et al. [15], where they used Laser Projection-Sensing Technology and proved the accuracy of ±0.04 mm. The method needs a unique laser sensor that must be placed in one spot so that the UAV can study the whole bridge at once. Also, it does not require sensitive installation and careful calibration. Still, there are several downsides: the first occurs when there is not a drone nest installation on the bridge so it requires physical travel of drone operator to the measurement site. The UAV are sensitive towards weather conditions and susceptible to rocking. Rocking can be reduced by use of damping system.

The method of structural elements segmentation, benefits form the use of Radon transform properties. The most significant advantage of our approach is proper shape detection, which can be described by basic geometric shapes. In our opinion, this solution is ideal for analysing structural elements that most often correspond to such forms. As we mentioned in Section 2.2, Abdel-Qader et al. [16] use region growing-based approach to segment thermal images for noninvasive evaluation of bridge decks. We think that this algorithm is useful for large colour spectrum images, such as thermal images. In our case, we deal with images that represent structural elements, which is not characterised by a broad colour spectrum. They are less or more uniform. An excellent example of image segmentation was presented by Duarte A. et al. [49] for biomedical purposes. They used Region of Interest (ROI) based techniques for segmentation of medical thermography images. The authors point out that the biggest challenge is to recognise shapes that cannot be described by simple shapes, such as rectangle or circle. ROI is mostly used for such purposes. They proposed a method in which we can define ROI shape before algorithm execution. For medical studies, such a way seems to be useful. However, structural elements that we try to classify are regular in shape, in our case beams are longitudinal.

The next topic is the rivet detection and displacement tracking. One of the criterion that we adopted for our solution is the ability to recognise the geometric shape of the rivet head, which is round. Using the method we proposed, we could detect most of the rivets, and if some rivets were missed, determine its probable location. We correctly determine the position of rivets, but it turns out that when it comes to detecting rivets, the solution is extremely susceptible to image rotation, which was demonstrated by our robustness analysis. The conclusion is that segmentation is a very important step, as well as a large sample base, the number of frames per second that the camera can record. The more image samples, the more likely all rivets will be detected. There are other similar solutions, which seem to be well suited to perform this task. Rivet detection can also be performed by the previously mentioned ROI method [49] as rivet heads are circular. A different approach where ROI was also used is presented in the previously mentioned paper by Feng et al. [11]. Another interesting approach is presented by Borza et al. [50], where they built a system that automatically extracts the position of the eyeglasses, shape and size of the frame lenses in facial images. They presented an excellent example of using the edge detection algorithm, but it is very unusual because it is based on the use of probabilistic techniques. First, they built a model for representing the shape of the eyeglasses lens by Fourier descriptors. Then, they generated the search set, starting from a small number of representative lens shapes, with use of Fourier morphing. Finally, they used a lens contour extraction algorithm utilising a multi-stage Monte Carlo sampling method. They achieved for an eye detection the following results 98% detected, 2% false negatives and 0% false positives, as for a lenses contour it was 92.3%, 2.5% and 5.2%, respectively. In our case, we analysed 12 images of the segmented steel cross-braced beam, achieving on average 97.2% correctly detected rivets, 1.3% incorrectly detected rivets and 0.9% undetected rivets.

The last topic is rust detection. The efficiency of the model we built is high, and it detected 96% of the surface where rust occurs. The weakness of our method is impracticability of the detection of corrosion that occurs under the surface of the beam coverage. Another downside that can harm the effectiveness of the technique is a presence in the image frame of objects that have the same colour scale as colours that we used to create our model. This situation can occur if the bridge builders use the specific paint that coincides with the colour palette used, or if there are many people on the bridge, the colour palette we use is similar to human skin, e.g., a pedestrian communication route has been designed on the deck. Mentioned in Section 2.2, Sharifzadeh M. et al. [17] developed a method for detection and classification of steel surface defects, such as coil breaks, holes, scratches and rust, where they achieved accuracy of 88.4%, 78.0%, 90.4% and 90.3%, respectively, with a dataset of 250 steel defect images. They adopted an approach in which they try to adjust their solution to a specific defect. Rust defects are detected by thresholding, with the use of several algorithms, as follows Maximum Entropy Sum Method, Entropic Correlation Method, Renyi Entropy. They achieved the best results by using Renyi Entropy algorithm. However, we think that such board number of different algorithms may cause difficulties in the application of this method in practice. The study also does not clearly explain which algorithms are better and in which situations. Acosta et al. [51] presented an algorithm for detection of rust areas using digital images. They used a fascinating method: with the help of Perlin Noise algorithm, they simulated what rust would look like on images representing steel. Procedural-generated rust was applied to metal images and taught the Bayesian classifier to recognise them. In our opinion, the results should be discussed more thoroughly, but the method itself is innovative. We believe that machine learning algorithms are an excellent way to develop the problem we describe. In future research tasks, we will consider using such solutions ourselves.

## 5. Summary and Conclusions

We presented the framework for noninvasive diagnostics of bridges using Unmanned Aerial Vehicles (UAVs). Our procedure consists of image acquisition, preprocessing, segmentation, extraction of features and quality assessment. We described the data collection process using UAVs, including the optimal flight path. We listed several factors that may affect the acquisition process, such as adequately selecting the focal length of the camera, adequate close-up of details, ensuring the orthogonality of the optical axis to the examined object and eliminating unfavourable external factors such as atmospheric conditions. The operator should be notified of the initial verification of the image quality with a maximum measurement error of 1 mm. Therefore the cross-brace of the beam 220 mm wide should be represented by at least 220 image points. The rest of the procedure involves using the Radon Transform to segment the image and extract the steel cross-braced beams for further analysis. In our opinion, by tracking changes in the position of rivets in the local and macro-scale, we can determine probable failure, progression rate and its nature. For the considered case of cross-braced Fitzpatrick bridge, we were able to detect the majority of rivets, on average 97.2% and also determine the correct probable location of unclassified rivets. We performed the robustness test of our method, and we found out that it accurately detects the position of the rivet, but has issues with identifying rivets in rotated images. We conclude that to use our method correctly, one cannot skip the previous step related to beam segmentation, and also a more significant number of image samples analysed will benefit the final result. The next major issue, which indicates a poor condition of the considered steel bridge, is excessive corrosion. To tackle that task, we developed a method to determine potential external corrosion centres and their distribution. To identify the corroded areas, we built a rust colour model in the chrominance space based on 15 random samples representing the corroded area. The use of such a model allowed for the correct classification of 96% of rusted areas. Our solution is the framework and it needs validation in the form of long-term studies. Potentially, it can be used as a tool to perform constant monitoring of steel crossed-braced bridge structures and to warn against a progressing failure or deteriorating structural condition. In further research, we want to conduct long-term field tests and also develop methods for segmentation of structural elements for other types of bridge structures. In subsequent works, we would also like to create a model for detecting visible signs of structural fatigue and implement machine learning.

## Figures and Tables

**Table 1:** Deviations of the Euclidean distance of the rivet heads from the reference point depending on the rotation of the image.

Parameter	θ = −1.5°	θ = −1.0°	θ = −0.5°	θ = 0.5°	θ = 1.0°	θ = 1.5°
N1	−0.385	0.149	−0.300	1.164	0.728	−0.015
N2	0.705	0.397	0.231	0.309	0.152	0.059
N3	−1.181	−0.400	-1.103	−0.741	−0.343	−0.630
N4	−0.160	−0.355	−0.452	0.134	−0.830	−0.546
N5	0.047	0.033	−0.247	1.046	0.777	1.166
N6	0.588	0.852	0.441	−0.505	-1.064	0.408
N7	−0.792	−0.011	−0.456	0.314	0.848	−0.443
N8	−0.930	-	0.458	−0.479	−0.828	−0.828
N4	−0.160	−0.355	−0.452	0.134	−0.830	−0.546
N5	0.047	0.033	−0.247	1.046	0.777	1.166
N6	0.588	0.852	0.441	−0.505	-1.064	0.408
N7	−0.792	−0.011	−0.456	0.314	0.848	−0.443
N8	−0,930	-	0.458	−0.479	−0.828	−0.828
N9	−0.721	0.455	0.136	0.109	0.431	0.513
N10	0.448	−0.469	−0.380	−0.462	−0.773	-
N11	-	-	0.488	0.380	-1.211	-
N12	-	-	-	−0.779	0.649	-
N13	-	-	−0.374	−0.752	0.391	-
N14	-	-	−0.149	−0.211	−0.433	-
